# Caregivers’ Experience of End-of-Life Stage Elderly Patients: Longitudinal Qualitative Interview

**DOI:** 10.3390/ijerph19042101

**Published:** 2022-02-13

**Authors:** Eliza Lai-Yi Wong, Janice Ying-Chui Lau, Patsy Yuen-Kwan Chau, Roger Yat-Nork Chung, Samuel Yeung-Shan Wong, Jean Woo, Eng-Kiong Yeoh

**Affiliations:** 1 Centre for Health Systems and Policy Research, The Jockey Club School of Public Health and Primary Care, Faculty of Medicine, The Chinese University of Hong Kong, Hong Kong, China; janicelau@cuhk.edu.hk (J.Y.-C.L.); patsychau@gmail.com (P.Y.-K.C.); rychung@cuhk.edu.hk (R.Y.-N.C.); yeungshanwong@cuhk.edu.hk (S.Y.-S.W.); yeoh_ek@cuhk.edu.hk (E.-K.Y.); 2 The Jockey Club Institute of Ageing, The Chinese University of Hong Kong, Hong Kong, China; jeanwoowong@cuhk.edu.hk

**Keywords:** end of life care, EOL, end stage care, long term care, informal care, caregiver, stress, healthcare system, longitudinal qualitative

## Abstract

Objectives: This study seeks to provide an understanding of the changing experiences in caregivers of end-of-life patients in Hong Kong through exploring their caregiving journey. Methods: Using longitudinal individual qualitative interviews, a total of 14 caregivers of community-dwelling elderly patients receiving end-of-life care were recruited between 2015 and 2016. A series of in-depth interviews and observations were conducted in 14 cases during the end-of-life journey. Results: A thematic analysis revealed four sequential experiential stages, abbreviated as “CAPE” that caregivers confronted: Stage 1 Certainty, (1a) lack of certainty regarding the progression of decline at the end-stage of life (1b) feelings of despair as patients’ function decreased; Stage 2 Ambivalence, (2a) feelings of ambivalence after decisions were made regarding EOL care, (2b) struggle over care responsibility within families; Stage 3 Perturbed, (3a) varied in quality of EOL care, (3b) depressed mood arisen from frequent exposure to the suffering of elderly patients; and Stage 4 Expectation, (4a) losing the caregiving role as patients showing signs of imminent death. Conclusions: These findings increase our understanding of caregivers’ in-depth experience over time that arise within the structural context of end-of-life care. Our data highlights the need for end of life related knowledge and information, provision of a caring atmosphere and communication, and professional-led detachment in creating caregiving-friendly service in healthcare system, thus as to provide support and alleviate stress for caregivers with their critical responsibility and role during the course of end-of-life care.

## 1. Introduction

Medical breakthroughs have resulted in longer life expectancies than were observed in the previous century. However, an increasing number of people are living with chronic diseases that require long-term care. As a result, care for the elderly involves not only professionals in a conventional hospital setting but also informal caregivers (e.g., family members, relatives, domestic helpers, or friends) in non-hospital settings. Informal care can complement formal care by increasing the quality of care a patient receives, in addition to decreasing the burden of such care in the health care system [1]. Worldwide, approximately 75−95% of patients die in an institutional setting, but this is changing toward an ethos that favours “aging in place”. Hence, the implementation of home care is being encouraged by specialists in palliative care and geriatric services. However, these developments may presuppose the readiness of families to deliver end-of-life (EOL) care at home, which hinders achieving a sufficient level of quality and standard of care. Many studies have documented that home care is not without its stressors on caregivers, such as anxiety, depression, fatigue, deterioration in family relationships, a decline in physical health, and social isolation [2,3,4]. Overall, in recent years in various countries, mental [5,6,7], physical [6], financial [8], and social stressors [9,10] have been frequently reported by family caregivers of dying individuals, signaling the importance of seeking to better understand their manifestation. 

Similar to other developed countries, Hong Kong is facing challenges associated with an aging population [11]. The population of individuals in the city who are at least 65 years old increased from 7.6% in 1980 to 16.1% in 2015, and projections now show that the elderly will account for approximately 35.9% of the entire population by 2064 [11]. Despite efforts made advocating “aging in place”, Hong Kong has one of the highest institutionalization rates in the world, at 6.8%, with other countries ranging from 1.0% to 5.4% [12]. One of the possible reasons for this is the inability of families to shoulder the responsibility of such care [13]. Therefore, in order to support family members to provide care in the community, this study aims to increase our understanding of the caregivers’ experience along the end of life journey to strengthen interventions and further enhance policy formulation to support “aging well in place”.

## 2. Methods

### 2.1. Study Design 

This study employed a longitudinal qualitative approach using a series of in-depth interviews with observations of patients approaching EOL stage and his/her family caregiver. A total of 14 cases were recruited thus to offer a way to explore a range of variables within a real-life setting [14]. We adopted a longitudinal study to capture changes in experiences and views through focusing on how and why these experiences changed in caregivers over time, particularly in the context of patients at EOL stage whose condition may vary from the last few months to the last few days. Furthermore, this approach has the advantage of collecting an individual’s views over a period of time compared to a single interview [15]. Our participants were followed over a period of 6 months or stopped when patients died, conducting interviews at 1-month intervals for up to 6 times. The interviews were the primary source of qualitative data and were supplemented with observation notes of events, including interviewees’ facial expressions and interactions with their surroundings, strengthening the validity of our findings. 

### 2.2. Setting

Our cases were purposefully sampled and recruited from the geriatric wards of 4 selected hospitals located on Hong Kong Island, Kowloon, and the New Territories, where EOL care services for patients at risk of dying were provided. EOL care programs were in place at 3 of the studied hospitals that provide services for symptom control, and direct admission to designated beds (at convalescent setting) through bypassing emergency departments during office hours were provided. The remaining hospital was in a stage of preparation for EOL program, yet advanced care planning (ACP) discussions could still be conducted and Do-Not-Attempt Cardiopulmonary Resuscitation” (DNACPR) forms could still be signed with patients with a prognosis of EOL. Patients who are clinically assessed with a prognosis of EOL refer to the prediction of patient death within 6–12 months according to the Gold Standard of the United Kingdom [16]. Patients recruited from our study settings were usually receiving residential care at old age homes, which are either run by private owners or subvented by the government and with a minority residing at their own home, living with their family members.

### 2.3. Samples

Due to the exploratory nature of this study, purposive sampling with heterogeneity was used to enhance the richness of data in the longitudinal qualitative method. Potential participants were initially identified by hospital nurses, who briefed the study, and participants’ rights and information were further explained by our researcher, via phone, during the period from December 2015 to November 2016. To attain a broad understanding of the caregiver experience, all recruited cases representing a mixture of demographic factors were included. Inclusion criteria were that all patients were of Chinese ethnicity, and at age 65 or above, had caregivers who were identified as the patient’s guardian or the primary family caregiver and received EOL care in the public hospital.

### 2.4. Data Collection 

Participants who expressed interest in participating provided written consent before our interviews began. Since the interviews might potentially touch on sensitive and ethical topics, the caregivers were interviewed by the same interviewer (JYC Lau), who has extensive experience in conducting qualitative studies. Interviews were conducted in separate rooms of the studied hospitals, old age homes, or patients’ homes. They were audio-taped, and each lasted for approximately 45 to 90 min. We developed our interview guide with semi-structured and open-ended questions, which encompassed our inquiry into stress in EOL caregiving. Since the patients’ medical condition changed over time, caregivers might in tandem experience their work differently. Using longitudinal qualitative methods allowed us to understand how the caregiver’s experience changed or how they coped over time. The following 3 key questions were asked to draw out insights: “how have things been for you since your family member or relative approached EOL?” “what was the experienced stress/burden/challenges?” and “what support have you received that helped to relieve your stress/burden?”. These questions were posed depending on the unique circumstances of each dyad of patients and caregivers. The privacy and confidentiality of the participants were protected by maintaining their anonymity during interviews. The observation was conducted at locations of patients’ residences, including homes or old age homes, and at the convalescent ward or acute ward during their hospital stay. We observed verbal and behavioral interactions between patients, family members, healthcare professionals, and nursing staff at varied settings that may signify the source of stress among family caregivers. 

### 2.5. Analysis

Audio data was fully transcribed verbatim into Chinese by the researchers; they were then crossed checked by the team members (ELY Wong, N Kiang) to ensure accuracy. As data collection began, our researchers familiarized themselves with the first 5 transcripts and independently conducted open coding on them using NVivo software. The team members reviewed, discussed, and agreed with the initial coding categories that were then applied consistently for the rest of the transcripts. Following open coding, researchers conducted axial coding, i.e., identifying relations of stresses and challenges in relation to particular behaviors, events, and people. A grounded theory approach was used, through which we examined, reviewed, and reached a consensus regarding themes. The themes were presented by thematic analysis in the following sections, including representative supporting quotes. Research rigor was ensured through an iterative process of data triangulation sourced from our interview transcripts, observation fieldnotes, and stages of the EOL care program available at the studied hospitals. This process was used to strengthen data validity by capturing congruence or discrepancies in our data. Our authors who took part in reviewing and examining data provided multiple perspectives, thus avoiding research bias that may result if the study was conducted by a single investigator. 

## 3. Findings

A total of 17 caregivers were initially approached, and 14 of them were successfully recruited and interviewed. In all, 51 interviews were conducted. The reasons given for declining participation included two caregivers who could not participate due to time constraints and one caregiver of a patient who passed away as soon as recruitment was completed. Thematic analysis was conducted in a total of 51 interviews among 14 participants. The team spent nearly a year (14 December 2015–26 December 2016) conducting interviews, and the average number interviews was 4 (range: 1–5) to provide data saturation. The demographic characteristics of the patients and also their caregivers (study participants) are shown in Table 1a,b, respectively.

**Figure 1:**
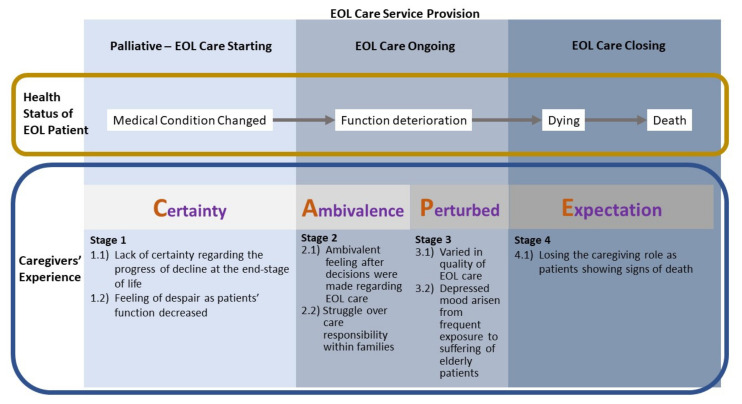
Thematic map of four stages “CAPE” reflecting caregivers’ challenges along EOL journey.

After a series of in-depth interviews with the prospective longitudinal approach, four stages—Certainty, Ambivalent, Panic, and Expectation (CAPE)—were identified to reflect the stress and challenges experienced by the caregivers along their EOL journey. Seven themes associated with the four stages were identified as sources of stress: (1) deteriorating health conditions; (2) frequent exposure to individuals at the EOL; (3) EOL care decisions; (4) quality of EOL care at institutions and; and (5) responsibility within families. A thematic map that represents these findings is shown in Figure 1, and the related information is mentioned in the following paragraphs.

### 3.1. Stage 1 Certainty

#### 3.1.1. Lack of Certainty Regarding the Progress of Decline at the End-Stage of Life

The majority of our participants regarded the uncertainty in EOL prognosis as a stressor to providing end-of-life care. They were uncertain about the coordination of EOL care and the expected period of care provision. One of the participants reported emotional difficulties when being notified by nursing home staff of her husband’s hospital admission since the situation might escalate suddenly and she did not feel she had a clear understanding of the pathway of EOL care.

I used to go along with him when he was required to be sent to the hospital by ambulance, but recently, I could not. I felt like I was out of control!…Everything is fast…sudden. Because of the unknown… I felt scared whenever I received the phone call…

(Case 7)

#### 3.1.2. Feeling of Despair as Patients’ Function Decreased

Although the majority of participants in this study have been providing care to the elderly for a period of time, recognizing signs of deterioration remained a significant source of stress. Some participants revealed feelings of despair due to care recipients’ decreased physical and cognitive functions, resulting in loss of control, dignity, and personhood. One caregiver reported having unstable moods and often cried when she was home alone:

My husband has lost both emotional and physical feelings because of the pain. The skin on his ear is no longer intact. There are wounds here and there with reddish coloured skin patches. Every time I return home after visiting him at the nursing home, I start to cry…!

(Case 7)

Comparisons have been made by some participants between caring for infants and caring for individuals at the end of life. However, while the former was seen as a joyous task as their functions increased, the latter was seen as source of melancholy as their functioning deteriorated. A son initially stated he was undisturbed by his mother’s deteriorating state of health:

I am feeling ok, this (deterioration) is part of the life process. What is important is that if she really has to go, she can pass away comfortably. It really doesn’t matter… it doesn’t concern me.

(Case 4)

However, he admitted that he felt pessimistic in the final round of interviews:

When you look after a baby, you are full of joy because (s)he is growing. However, when you look after your mother, you are very sad as you see her deteriorating day by day. Therefore, it’s different… you’re sorrowful while looking after your mother!

(Case 4)

### 3.2. Stage 2 Ambivalence

#### 3.2.1. Ambivalent Feeling after Decisions Were Made Regarding EOL Care

While some of the participants in this study stated that they felt more settled after care plans were made for their dying relatives, some reported feelings of ambivalence. A series of questions related to the care plan was raised among participants, including whether it was necessary to start an antibiotic treatment regimen, to start nasogastric feeding, or to have blood tests performed. The following excerpt reflects the difficulties faced by a daughter in deciding whether or not to enrol her mother into the EOL care program, as she was concerned that her intentions to end invasive life-sustaining treatment and relieving her mother’s suffering would be misinterpreted as a lack of filial responsibility:

After all, I said to her, “We’re not trying to send you away (by causing death), but we just do not want you to suffer!” I cried immediately after I spoke to her!… When I looked at her, I wish she could leave (the world), but I would not want to because I miss her. I am so ambivalent!

(Case 5)

Despite having signed a Do-Not-Resuscitate (DNR) form, one participant had concerns about whether or not to notify healthcare providers at acute settings regarding the form, as she was worried that healthcare providers would not provide attentive care to her mother:

After all, I’m still concerned whether I should present the form immediately (when my mom is admitted to the hospital)… in case my mom has low blood pressure, I wonder whether they (healthcare providers) would ask physicians if there is anything they could do or whether they might just simply leave her because we had signed the DNR form.

(Case 14)

#### 3.2.2. Struggle over Care Responsibility within Families

Most participants in this study revealed experiences of stress while caring for family members at the end stage of life. Some of the female caregivers who had jobs felt that they were obligated to provide care that optimized the health conditions of their family members and, therefore, felt the need to juggle between their roles as caregivers and paid employees. One caregiver felt that she was expected to be attentive to the needs of her husband and provide care around the clock, even though he resides in a nursing home, as this was regarded as a women’s obligation. She preferred to remain vigilant by accompanying her husband at all times instead of engaging in social activities:

My friends asked me to relax and take a 1–2 days tour during which we could have some food. However, I would not feel at ease because what if something happened to him while I was away!… I would feel guilty for my dereliction of duty….

(Case 12)

Furthermore, more than half of our participants reported a lack of instrumental support from their siblings and/or family members that often led to sadness and loneliness. This sense of loneliness was driven by variations in perception of care responsibility, with some family members perceiving care as a “meaningful affection” and others viewing care as “burdensome”. The decision regarding where care was provided was reported to be especially stressful for the majority of the family members in this study. Deliberations between the desire for parents to live well and families’ capacity to cope with their needs often led to sadness, anger, disappointment, and feelings of being misunderstood. This resulted in divergence in care involvement among family members. For example, a daughter who resigned from her job to look after her parent for more than 10 years found it difficult to ask for assistance in providing care. She felt that asking for assistance would be perceived as burdensome and unrealistic to her siblings:

When I felt sick and could not even get out of bed, I still needed to get up anyway to take care of my mom! I felt very helpless at that moment but, I could not tell my brothers and sisters. I could not make any complaints to them.

(Case 1)

Similarly, although most care recipients reside in nursing homes, the level of care among caregivers did not differ. While the notion that “institutional care is a way to alleviate the burden on the family” was upheld by their siblings, some caregivers expressed a mixture of anger and disappointment that family members were unable to collectively share the responsibility:

Yes, I do have expectations, but I ended with disappointment… oh well, just let them be, they’re adults! Everyone has a family, and everyone has a job! I always tell myself that I’d do whatever I can. I would not care about others.

(Case 5)

### 3.3. Stage 3 Perturbed

#### 3.3.1. Varied in Quality of EOL Care 

Those whose parent or spouse resided in privately owned nursing homes reported chronic strain more frequently than those living in publicly-funded nursing homes and communities. In particular, low staffing standards and lack of compassion led to exhaustion in caregivers. Participants found that making daily visits was the only solution to ensuring that their parents were cared for with dignity:

All nursing staff are employed from mainland China, and they have inadequate knowledge and very little compassion… For example, my helper told me that the bathroom was very dirty because the elderly sit on commode chairs when they shower, which meant that they often urinate at the same time… I cannot complain because after all, my mom requires their care. So I’m in a very difficult position.

(Case 3)

Furthermore, family members were irritated that healthcare professionals did not provide person-centred care to their family members. The following excerpt illustrates the feelings of a daughter, who was upset about the decision to prematurely discharge her parent from the hospital, as it led to hospital readmission that was potentially preventable:

My mom was readmitted to the A&E within less than 24 h, and because you are admitted to A&E again (instead of a convalescent hospital), they will treat it as a new episode. She had to go through the tests again, including X-ray, blood tests and other investigations, and this actually made her suffer!

(Case 3)

Another participant reported that she had been in repeated conflicts with healthcare professionals, including physicians, speech therapists and occupational therapists due to the lack of coordination in the advance care plans across the time and disciplines:

The doctor even said to me, “I do not recognize this (EOL) programme. I’m here to cure patients and I do not think that your mom is sick enough to die soon!”

(Case 9)

#### 3.3.2. Depressed Mood Arisen from Frequent Exposure to Suffering of Elderly Patients

In addition to observing family members’ deterioration, frequent exposures to individuals at the EOL also had an emotional impact on our participants:

In fact, of those admitted to this ward…many of the elderly passed away…and most of them were very frail… They neither ate nor took care of themselves. In addition, they moaned sometimes. Seeing these things made me feel really uncomfortable.

(Case 3)

Our observation in the field notes also recorded the following regarding the environment of an EOL ward while visiting a care recipient:

While feeding her mother, our participant looked across the room at an old lady who was breathing heavily, with no one beside her. The daughter then turned around and looked at the researcher and said, “She’s going to die soon! Isn’t it depressing to see all these people around?”

(Case 9)

### 3.4. Stage 4 Expectation 

#### Losing the Caregiving Role as Patients Showing Signs of Death

Levels of stress associated with EOL care management varies according to the progression of the illness. Many participants reported a sense of helplessness as their caregiving role diminished, because the tasks they had once performed no longer met the needs of care recipients. For example, a daughter reported that she felt useless when she realized that her mother was gradually becoming unresponsive, but there was nothing she could do for her:

I’m not sure whether my mom knew what she was doing. I want to buy her food, but she cannot eat. I wish to have a chat with her, but she cannot speak… I feel so much pain for her!

(Case 5)

Some caregivers expressed grief and wished that they could reconnect with their dying relatives as they observed signs of death:

As I observed that her condition was getting worse, I felt that she was leaving us soon. I got more sensitive to everything surrounding her. They (memories) come and go like a flash!… There are things I wish I could have done with her…!

(Case 3)

## 4. Discussion

This study contributes to understanding the changes in caregivers’ experiences throughout the EOL journey. Our findings revealed a series of potential stressors and psychological responses: “C”ertainty, “A”mbivalent, “P”erturbed and “E”xpectation (CAPE) in the EOL care journey. Inadequate knowledge and in caregiving capability were potential stressors in EOL care that led to doubt in caregiver preparedness. This includes knowledge on the progression of decline in health and the provision of EOL care. Moreover, inadequate knowledge and information also affected family caregivers’ perception of being in control and their ability to make advanced care plans, as findings revealed feelings of uncertainty as well as ambivalence among caregivers following the making of EOL decisions. End-of-life caregiving requires new knowledge and mindset because the duration of end-of-life caregiving facilities learn new skills or gain confidence in abilities and certainty in decision making [17]. Previous studies have revealed that making decisions to accept or reject life-sustaining treatments for family members are seen as difficult and stressful [18,19,20,21], as “non-resuscitation” is often perceived as “giving up” [22,23,24]. These findings, in addition to previous evidence that information and training interventions can lead to positive experiences for caregivers [25,26], suggests the need for training and education for caregivers and family members. 

Besides the inadequate knowledge of health status and service provision in EOL, “exposure to suffering due to lack of knowledge” to individual family members and patients led to panic among caregivers. This finding is consistent with previous studies that reported that caregivers experience similar emotions in response to care recipients’ suffering [7]. Self-efficacy is highlighted as one of the key dimensions posing the greatest threat to caregivers’ well-being at the end of life, in which low levels of confidence in providing care may lead caregivers to ignore their own health and well-being while managing caregiving responsibilities [27]. Patients are not sole care recipients in an EOL care provision, caregivers’ needs are also important as they are both co-caregivers in a formal care system and co-care recipients with patients. Informal caregivers play an increasingly important and active role in supporting care-dependent patients. In EOL, a caregiver is a particularly important delegate and proxy of the patient who helps them express their preferences and makes decisions. There is a need to adopt patient-reported experience measures to improve the care process by understanding the experiences of both the patients and caregivers. Some aspects influencing care experience were important for participants to obtain a positive outcome, such as noise, lighting, caring atmosphere, and providing sufficient time for open communication [28,29,30]. Knowledge and reinforcement allow the caregiver to anticipate the patient’s needs; their certainty is heightened, and they have more confidence to continue with their caring role. 

In Chinese culture, family solidarity and filial piety play a major role in providing care to the elderly [31,32,33]. While daughters are the main informal caregivers, this study highlights weak family solidarity in the provision of EOL care. Participants revealed the lack of support from family members, specifically siblings, resulting in stress and the burden of caregiving. These findings suggest the need to support informal caregivers in the community. In the UK, for example, caregivers are supported by different social support systems, including MacMillan nurses who pay regular visits to patients, reducing family members’ burden [34]. In Hong Kong, a community nurse is provided to support those older adults at high risk of hospital readmission. The findings of this study provide support for the need to extend the outreach nursing support for the EOL case, taking the UK as reference, to provide education, psychological support, and care to enhance family solidarity as well as reduce caregiver’s burden. Healthcare professionals play a significant role in caregivers’ experience at the end of life because professionals’ support can take the pressure off the shoulders of caregivers and can be a gateway to obtain resources [35,36,37]. 

Furthermore, some of our participants manifested a sense of dedication to care as they exhibited a preference for part-time work that allowed more flexible working hours to be a “responsible” informal caregivers at hospital and community settings. Caregivers reported unpleasant experiences during the phase of dying and death, mainly due to feelings of incapability and loss. These feelings made it difficult for caregivers to detach themselves from their expected role and face the longing and emptiness they experienced following the loss of their family member [37]. Reinforcing this finding, our study highlights the perception by these caregivers that they are unable to perform their expected role due to the poor progression of illness at the terminal stages of EOL care. In spite of sacrificing their careers and personal life, they become incapable of continuing in their role as caregivers. This experience of role withdrawal toward the end of life is inextricably linked to the unexpected developments and struggles in caregivers. Participants indicated that having care routines that fit with their role and ability was essential. Thus, it is a very challenging process for caregivers to adapt and reconcile with “normal life”. Engagement of caregivers in different forms and ways of EOL care service provision is critical to building trust, openness, empathy, and acceptance [27,38,39]. Emotional support and reinforcement from healthcare professionals’ are essential to enable caregivers to detach from their caregiving responsibility and heighten their bonding with the patients on a spiritual level. In addition to the healthcare workers, enhancement of the medical and nursing curriculum is important to build a caring culture and empathetic communication in healthcare [40,41]. This can reduce the psychological impact by shifting from tangible care to intangible care stages between caregiver and family members. In addition to knowledge, compassionate care in the context of the end of life is particularly important to increase caregiver’s satisfaction of service, produces a positive effect on well-being, and soothes grief, including loss of a family member, being away from a caregiving role, change in identity, etc. 

### Strengths and Limitations of This Study

The longitudinal case design of this study using purposive sampling allowed us to obtain rich narratives to perform a critical qualitative analysis of the stressors experienced by family caregivers across the journey of EOL. However, one limitation related to the generalization of our data should be noted. Because we required a substantial time commitment to join this longitudinal study, one caregiver could not participate. This may imply that other caregivers, including those who are under the most stress, perhaps because of their multiple roles, may not have been approached by the nurses during the recruitment phase. Nevertheless, we are the first to use a longitudinal case study design to explore the issues associated with and the rationales that underlie a person’s caregiving role in an EOL care context in the local region. Lastly, the data collection and analysis were completed in mid-2017. Thus, the gap between project completion and publication may hamper the validity of the findings. However, our results of this study enrich the body of evidence about the changes in experiences of caregivers of patients receiving EOL care. It, therefore, warrants future research to evaluate whether supplementing care with other elements, such as social care and public education, could reduce stress in family caregivers and strengthen cohesion among family members.

## 5. Conclusions

This longitudinal case study represents the first attempt to gain a deep understanding of the stressors experienced by caregivers during their care journey in an EOL care context. Our findings reveal that the quality of EOL care impacts the well-being of caregivers, implying that the support provided to these caregivers during their care journey is currently inadequate. Furthermore, we found that the general preference of individual caregivers to provide comfort care and dignity was hindered in homes for the elderly and the hospital settings. It would, therefore, be valuable for future studies to explore views about “quality of EOL care” among nursing staff, healthcare professionals, and the broader general population in this local region. In addition, attention should be paid to gaining insight into how individual care experiences and preferences related to EOL care processes. Thus, we lay an important foundation that should guide the design of EOL care models to more effectively respond to the needs of caregivers who play a critical role in co-caring for patients in contemporary health systems.

## Figures and Tables

**Table 1:** (**a**). Demographics of included patients (*n* = 14 *). (**b**). Demographics of caregivers (*n* = 14 *).

(**a**)	
	**N (%)**
**Age**	
70–79	4 (28.6)
80–89	4 (28.6)
90+	6 (42.9)
**Gender**	
Male	2 (14.3)
Female	12 (85.7)
**Marital Status**	
Single	1 (7.1)
Married	5 (35.7)
Widower/ Divorced	8 (57.1)
**Place of residence**	
Home	3 (21.4)
Nursing home	11 (78.6)
(**b**)	
	**N (%)**
**Age**	
≤49	3 (23.1)
50–59	7 (53.8)
60+	3 (23.1)
**Relationship with care recipient**	
Wife	2 (14.3)
Daughter	10 (71.4)
Son	1 (7.1)
Godson	1 (7.1)
**Marital status**	
Single	5 (38.5)
Married	7 (53.8)
**Education level**	
Primary	2 (15.4)
Secondary	9 (69.2)
University	2 (15.4)
**Working status**	
Unemployed	1 (7.1)
Self-employed	1 (7.1)
Housewife	5 (35.7)
Employed	7 (50)

* The numbers did not add up to the total (*n* = 14) due to missing data.
